# Mineral Nutrition and Human Health and Disease

**DOI:** 10.3390/nu18030370

**Published:** 2026-01-23

**Authors:** Salvatore Minisola, James H. Swain

**Affiliations:** 1 Department of Medical and Cardiovascular Sciences, “Sapienza” Rome University, 00161 Rome, Italy; 2 Department of Nutrition, School of Medicine, Case Western Reserve University, 10900 Euclid Avenue, SOM WG-48, Cleveland, OH 44106, USA; jhs31@case.edu

Healthy nutritional intake is fundamental for the physiological development of organs and tissues and, later in life, for their optimal function. In this context, mineral nutrition, including both major elements (i.e., calcium, phosphorus, magnesium, potassium and sodium) as well as trace elements (i.e., iron iodine, zinc), is a major component of the preservation of a number of functions. These include those related to the skeleton, endocrine, and the haematopoietic system.

To provide couple of examples, inadequate vitamin D levels have detrimental effects on the skeleton through the insufficient absorption of both calcium and phosphate in the intestine [1]. The decrease in serum calcium levels is compensated by a secondary increase in parathyroid hormone secretion. Long-term consequences on the skeleton are well-known in terms of reduced bone mineral density, increased risk for fractures, and, depending on the severity and duration of vitamin D deficiency, osteomalacia.

Iron is not only important in the production and function of haemoglobin and myoglobin but is also an essential co-factor in many other processes, such as mitochondrial energy production and neurotransmitters synthesis. Individuals with iron deficiency are sometimes asymptomatic but may have a number of signs and symptoms as a consequence of reduced oxygen levels. Among these, fatigue and reduced exercise tolerance are prominent. Recent findings also highlight the negative effect of hypoxia on bone. Indeed, both skeletal and muscle tissue implement specific responses to hypoxemia [2]. It has also been hypothesised that oxygen-sensing can control osteocyte function, with possible implications for the ageing-mediated dysregulation of oxygen bioavailability. This can have a role in the pathogenesis of reduced bone mass with advancing age [3].

In the Special Issue of this journal, targeting “Mineral Nutrition and Human Health and Disease”, we provide ten contributions, both experimental and review studies, aiming to better understand current issues and offer balanced solutions. Various topics are discussed; however, in the modern era, a couple of observations need to be emphasised.

There is now a large body of evidence that the quality of our foods is deteriorating. Ultra-processed foods seem to have an increasing role in the pathogenetic mechanisms of chronic non-transmissible diseases [4]. The manuscript by Souza et al. [5] addresses these problems, showing how processed foods modify the Na/K ratios in manufacturing workers. In this context, it is important to refer to a recent paper published by Greatorex Brooks et al. Analysing the NHANES data, they showed an association between osteoporosis and osteopenia and the intake of ultra-processed foods as a proportion of total daily energy [6].

Finally, some papers in this series refer to the importance of the microbiota for adequate mineral nutrition. There is now substantial evidence that changes in gut microbiome with ageing may be instrumental in osteoporosis, a typical disease of ageing [7]. Future therapies should also consider this aspect to guarantee a healthier transition in the last decades of life.

Looking ahead, the future of research in the area of mineral nutrition should provide exciting new insights, especially with the introduction of new technologies and an increased understanding of the interplay between genetics and dietary intake. This Special Issue contributes to this effort, and the increased knowledge it provides promises to assist in the development of more precise recommendations, better prevention strategies, and innovative solutions to global nutrition challenges.
